# Long-Term Bed Rest Delays the Circadian Phase of Core Body Temperature

**DOI:** 10.3389/fphys.2021.658707

**Published:** 2021-05-10

**Authors:** Stefan Mendt, Katharina Brauns, Anika Friedl-Werner, Daniel L. Belavy, Mathias Steinach, Thomas Schlabs, Andreas Werner, Hanns-Christian Gunga, Alexander C. Stahn

**Affiliations:** ^1^Charité – Universitätsmedizin Berlin, corporate member of Freie Universität Berlin and Humboldt-Universität zu Berlin, Institute of Physiology, Center for Space Medicine and Extreme Environments Berlin, Berlin, Germany; ^2^INSERM U 1075 COMETE, Université de Normandie, Caen, France; ^3^School of Exercise and Nutrition Sciences, Institute for Physical Activity and Nutrition, Deakin University, Geelong, VIC, Australia; ^4^Charité – Universitätsmedizin Berlin, corporate member of Freie Universität Berlin and Humboldt-Universität zu Berlin, Center for Muscle and Bone Research, Berlin, Germany; ^5^Charité – Universitätsmedizin Berlin, corporate member of Freie Universität Berlin and Humboldt-Universität zu Berlin, Department of Internal Medicine and Cardiology, Berlin, Germany; ^6^German Air Force – Centre of Aerospace Medicine, Aviation Physiology Training Centre, Aviation Physiology Diagnostic and Research, Königsbrück, Germany; ^7^Department of Psychiatry, Perelman School of Medicine, University of Pennsylvania, Philadelphia, PA, United States

**Keywords:** inactivity, spaceflight, medical care, deconditioning, countermeasure, core body temperature

## Abstract

Spaceflight can be associated with sleep loss and circadian misalignment as a result of non-24 h light-dark cycles, operational shifts in work/rest cycles, high workload under pressure, and psychological factors. Head-down tilt bed rest (HDBR) is an established model to mimic some of the physiological and psychological adaptions observed in spaceflight. Data on the effects of HDBR on circadian rhythms are scarce. To address this gap, we analyzed the change in the circadian rhythm of core body temperature (CBT) in two 60-day HDBR studies sponsored by the European Space Agency [*n* = 13 men, age: 31.1 ± 8.2 years (*M* ± *SD*)]. CBT was recorded for 36 h using a non-invasive and validated dual-sensor heatflux technology during the 3rd and the 8th week of HDBR. Bed rest induced a significant phase delay from the 3rd to the 8th week of HDBR (16.23 vs. 16.68 h, *p* = 0.005, *g* = 0.85) irrespective of the study site (*p* = 0.416, *g* = −0.46), corresponding to an average phase delay of about 0.9 min per day of HDBR. In conclusion, long-term bed rest weakens the entrainment of the circadian system to the 24-h day. We attribute this effect to the immobilization and reduced physical activity levels associated with HDBR. Given the critical role of diurnal rhythms for various physiological functions and behavior, our findings highlight the importance of monitoring circadian rhythms in circumstances in which gravity or physical activity levels are altered.

## Introduction

The entrainment of the circadian timing system to the 24-h solar day is essential for various physiological and psychological functions. Circadian misalignment can impair cognitive and physical performance and lead to considerable negative long-term consequences on mental well-being and health (Thun et al., 2015; James et al., 2017; Xie et al., 2019; McGowan et al., 2020). Shift work, transmeridian flights, and spaceflight can challenge the temporal adaptation of the circadian system because of misalignments between the timings of the central clock (suprachiasmatic nucleus within the hypothalamus), peripheral clocks (tissue and organ systems), and external cues, i.e., “zeitgeber” (Baron and Reid, 2014; Roenneberg and Merrow, 2016; Dibner, 2020).

An increasing body of research shows that non-photic stimuli related to specific behaviors such as sleep (Crowley et al., 2015), regular mealtimes (Lewis et al., 2020), social routines (Mistlberger and Skene, 2004), and exercise (Atkinson et al., 2007) can serve as important cues for the circadian entrainment. Whereas improvements in performance and health by positive interactions of exercise with the circadian system are gaining recognition (Lewis et al., 2018), the effect of reduced physical activity and immobilization on circadian rhythms has so far received little attention. In the clinical setting, bed rest is used to address various medical conditions, often as an initial or prophylactic treatment following a medical intervention (Allen et al., 1999). There is evidence that patients treated in intensive care units (ICU) are particularly vulnerable to disrupted circadian rhythms (Papaioannou et al., 2014; Oldham et al., 2016). Whether bed rest contributes to circadian disruptions in ICU patients remains to be determined because the patients’ circadian rhythms can be affected by environmental factors and medical conditions including illness characteristics, delirium, medication, altered light-dark cycles, and noise (Korompeli et al., 2017; Telias and Wilcox, 2019).

Space agencies have been using head-down tilt bed rest (HDBR) to simulate critical physiological adaptations that astronauts encounter during spaceflight (Hargens and Vico, 2016; Pandiarajan and Hargens, 2020). Given the highly controlled and standardized protocols of this ground-based spaceflight analog investigating healthy people as astronaut surrogates (Sundblad et al., 2016), they overcome several confounders typically associated with clinical studies. They could play an important role in elucidating the effects related to bed rest on the circadian timing system. Circadian rhythms tend to phase shifts in response to HDBR (Samel et al., 1993; Monk et al., 1997; Hurwitz et al., 2004; Mendt et al., 2017). However, these studies comprised small sample sizes and differed in duration, data collection schedules, and circadian variables. To verify whether prolonged bed rest induces a robust effect on the circadian phase, we combined data on core body temperature (CBT) profiles collected at two different study sites, each investigating the effects of 60 days of HDBR. This was possible because the methodological approach for determining the circadian phase of CBT was identical across both study sites. We hypothesized that the lack of physical activity and absence of postural changes (i.e., reduction of the diurnal variations in rest/activity and sleep/wake cycles) weakens the circadian entrainment as indicated by a phase delay of CBT.

## Materials and Methods

### Study Design

The experiments were performed as part of the following two 60-day HDBR studies sponsored by the European Space Agency: (1) the “2nd Berlin BedRest Study” (*BBR2-2*), carried out at Charité – Universitätsmedizin Berlin, Germany in 2007/2008; and (2) “Effects of a nutritional cocktail consisting of anti-oxidant and anti-inflammatory supplements to prevent the deconditioning induced by 60 days of antiorthostatic bed rest” (*Cocktail*) performed at the Space Clinic of the Institute of Space Medicine and Physiology (MEDES-IMPS, Rangueil Hospital) in Toulouse, France in 2017. The primary objective of these studies was to assess the efficacy of a specific countermeasure protocol to prevent muscle and bone loss (resistive exercise, *BBR2-2*; antioxidant/anti-inflammatory supplement, *Cocktail*) compared to a control group exposed to bed rest only. Details related to the study designs can be viewed elsewhere (Belavý et al., 2010; Arc-Chagnaud et al., 2020). The environmental conditions (i.e., HDBR without countermeasure protocol) were comparable across both study sites. Participants remained in six-degree HDBR for 24 h/day during the entire bed rest period. Physical activities were limited to a minimum and performed in a lying position, including eating and personal hygiene. Participants were accommodated in single or double bedrooms with windows. Room temperatures ranged between 20 and 25 °C, humidity between 30 and 50%, and normobaric atmospheric pressures between 1010 and 1025 hPa. Regular sleep-wake cycles were maintained by a non-temporal isolated and conventional day/night cycle (natural daylight, domestic light) with lights off at 11 pm and scheduled wake-up between 6:30 and 7:00 am. In addition, any activity that interfered with sleeping was not allowed. The diet was carefully controlled and served on a fixed schedule three times per day. During their leisure time, participants had access to various media (reading, games, video, radio, and internet), and could communicate with family and friends *via* phone or email.

### Experimental Design

To establish the effect of HDBR on circadian rhythms *per se*, data from participants receiving any intervention were excluded from the data analyses. The data collection schedules varied between studies. To pool the data across the two sites, we identified those time points that were characterized by acceptable overlap. No data on CBT profiles were acquired before or after HDBR in the *BBR2-2* experiment, limiting a comparison of CBT profiles at baseline and recovery. In the *Cocktail* experiment data collection during HDBR was performed in two sessions, i.e., on days 19 and 52 of HDBR. Next, we identified comparable time points in the *BBR2-2* study in the 3rd and 8th week of HDBR, which corresponded to data collections on days 21 and 49. We then extracted and limited our data analyses to these time points (days 19 and 52 for *Cocktail*, and 21 and 49 for *BBR2-2*) to ensure the comparability of the data collected at the two different sites, and strengthen the primary objective of the study. First, continuous CBT measurements performed during the baseline data collection phase of bed rest studies are characterized by varying levels of physical activity and postural changes, which may potentially jeopardize the data quality for determining circadian rhythms in CBT (Waterhouse et al., 2005). Second, the first weeks of HDBR are prone to various adaptations that may confound circadian effects associated with prolonged bed rest. For instance, stress markers are significantly elevated after one week of HDBR (Rai and Kaur, 2011). Likewise, symptoms of discomfort and sleep difficulties have been described during the first week of HDBR (Meck et al., 2009). Therefore, using the data of the third week of HDBR as a baseline was considered a robust reference that allows assessing the change of the circadian rhythm of CBT in response to prolonged HDBR.

### Participants

Nineteen healthy men (*BBR2-2*: *n* = 9, *Cocktail*: *n* = 10) participated in 60 days of bed rest and were not exposed to any countermeasure protocol during HDBR. All participants had no history or physical signs of chronic diseases, sleep disorders, psychological, neuromuscular, hormonal disturbances, or addictions (alcohol, medication, or drugs) before entering the study. Each study was approved by the local ethics committee (*BBR2-2*: Charité – Universitätsmedizin Berlin, *Cocktail*: CPP Sud-Ouest et Outre-Mer I in Toulouse). All participants were informed about the purpose, experimental procedures, and risks before giving their verbal and written informed consent.

### Data Collection

Core body temperature was estimated non-invasively from the skin surface using a dual-sensor heatflux technology (Gunga et al., 2008; Uth et al., 2016). Data were collected for 36 h with a sampling rate of 0.5 Hz and all recordings started in the evening. The heatflux-based approach for determining CBT overcomes several challenges associated with standard CBT recording techniques, including but not limited to invasiveness, discomfort, compliance, and hygiene issues. The methodology has been shown to be well accepted for continuous CBT recordings in astronauts before, during, and after spaceflight (Stahn et al., 2017). Details about the underlying biophysical model of the sensor are reported elsewhere (Werner and Gunga, 2020). Briefly, the sensor calculates CBT from two temperature sensors separated by an insulating disk with a known heat-transfer-coefficient. The sensor was positioned at the participant’s forehead above the right eyebrow underneath the hairline and connected to a low battery-powered mobile monitoring system (HealthLab System, Koralewski Industrie Elektronik, Hambühren, Germany). Data were stored on a replaceable flash disk within the monitoring system and transferred to a personal computer. In *BBR2-2*, the so-called *Double Sensor* was used to record CBT, whereas a newer generation of this sensor (Tcore™) was used in the *Cocktail* study (Drägerwerk AG & Co. KGaA, Lübeck, Germany). In contrast to the *Double Sensor*, the Tcore™ sensor consists of a flexible self-adhesive material, improving the fit to the skin at the forehead, and increasing comfort during sleep. Both sensors have been found to record CBT accurately in clinical settings (Kimberger et al., 2009, 2013; Soehle et al., 2020). Recently, the technology and the measurement site have been found to provide a reliable estimate of the rectal temperature rhythm during HDBR (Mendt et al., 2017).

### Data Analysis

#### Data Preprocessing

To ensure data quality, all 36-h temperature recordings were visually inspected prior to analysis. Artifacts related to hygiene activities and signal errors were manually excluded. Since the start of the recordings varied, the temperature profiles were centered, i.e., data from 10 pm to 6 am the day after (32 h) were extracted from the 36-h temperature records, and then averaged over 6-min intervals. To evaluate temporal changes in response to HDBR, temperature profiles were subjected to the cosinor analysis using a cosine curve with a period of 24 h (Cornelissen, 2014). All cosine fits confirmed the presence of rhythmicity in the 32-h temperature profile (*p* < 0.0001). Each time series was quantified by the fitted curve parameters mesor (i.e., mean of the fitted curve), amplitude (i.e., half the difference between highest and lowest value of the fitted curve), and acrophase (i.e., time of highest value of the fitted curve).

#### Statistical Analyses

Rhythm parameters (mesor, amplitude, and acrophase) were summarized as means and their nonparametric bootstrap 95% CI (5,000 bootstrap resamples) unless stated otherwise. After checking the normality and homogeneity of the residuals using visual inspection (quantile-quantile plots), differences between the 3rd week and 8th week of HDBR were assessed by a two-way ANOVA with *Time* as a within-subject factor (levels: 3rd week, 8th week) and *Site* as a between-subject factor (levels: *BBR2-2*, *Cocktail*). Effect sizes were reported as Hedges’s *g* and their 95% CI using bootstrapping (Kirby and Gerlanc, 2013). The level of significance was set at *α* = 0.05 (two-sided) for all testing. All statistical analyses and graphical illustrations were carried out using “R” version 3.6.1 (R Core Team, 2019).

## Results

Two out of the 19 participants did not complete the CBT sessions, and data of four participants (two for *BBR2-2* and *Cocktail*, respectively) contained less than 75% of the 32-h recording. These data were excluded so that the final data set comprised complete recordings for 13 participants (*BBR2-2*: *n* = 5, Cocktail: *n* = 8). The final temperature profiles contained 97% [91–100%; median (interquartile range)] of the data of a 32-h recording [*BBR2-2*: 95% (89–99%), *Cocktail*: 98% (94–100%)]. Baseline characteristics of the final participants are given in Table 1.

**Table 1:** Participant demographics at baseline.

	*BBR2-2*	*Cocktail*	*BBR2-2* + *Cocktail*
Age (years)	28.2 ± 5.8	32.9 ± 9.3	31.1 ± 8.2
Weight (kg)	79.4 ± 5.3	73.6 ± 7.6	75.8 ± 7.2
Height (cm)	177.0 ± 3.2	175.6 ± 5.1	176.1 ± 4.3
Body mass index (kg/m^2^)	25.4 ± 2.0	23.8 ± 1.9	24.4 ± 2.0

Data are means ± SD. No significant differences between the *BBR2-2* (*n* = 5) and the *Cocktail* (*n* = 8) study (all *p* ≥ 0.127, Wilcoxon’s signed rank test).

**Figure 1:**
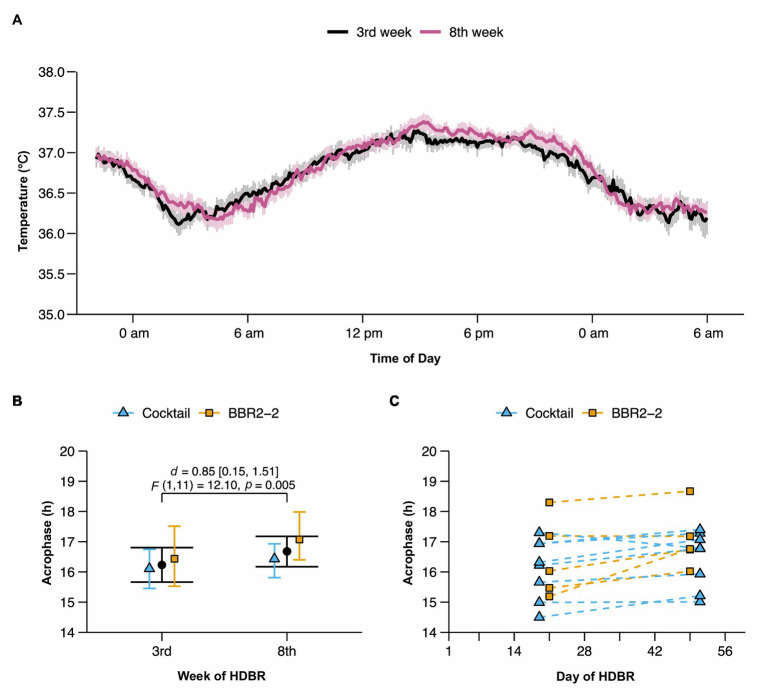
Changes in the circadian rhythm of CBT during head-down tilt bed rest (HDBR; *n* = 13 participants). **(A)** Average CBT profile in the 3rd week (black) and the 8th week (purple) of HDBR. Vertical bars denote SEs. **(B)** Mean phase and 95% CI are shown in black and for each study site in blue (*Cocktail*, *n* = 8) and yellow (*BBR2-2*, *n* = 5). **(C)** Changes in rhythm phase for each individual (dashed lines) during bed rest.

The average temperature profile in the 3rd and the 8th week of HDBR showed a clear daily rhythm with the lowest temperature in the early morning and the highest in the early evening (Figure 1A). Descriptive means and their 95% CI for mesor, amplitude, and phase for the 3rd and 8th week of HDBR are provided in Table 2. Mesor and amplitude remained unchanged. This was confirmed by the ANOVA model that did not reveal any significant main effects for *Time* and *Site* or their interaction (Table 3). We observed a phase delay from the 3rd [*M* = 16.23 h, 95% CI (15.68, 16.79)] to the 8th week [*M* = 16.68 h, 95% CI (16.16, 17.16)] of HDBR. This change was quantified by a large and significant main effect for *Time* on the phase [*p* = 0.005, *g* = 0.85, 95% CI (0.15, 1.51); Table 3]. No significant main effect was found for *Site*, nor was there a significant interaction between *Site* and *Time* (Table 3). Figures 1B,C highlight the study site related and individual changes in circadian phase during HDBR. The acrophase in the *BBR2-2* study was shifted compared to the *Cocktail* study (Figure 1B), which is likely to be attributed to the wake-up time scheduled 0.5 h later in *BBR2-2*. Yet, both studies elicited similar phase shifts (*BBR2-2*: 37.8 min after 29 days (~1.4 min/day); *Cocktail*: 19.2 min after 32 days (~0.6 min/day); total group: ~0.9 min/day).

**Table 2:** Effect of head-down tilt bed rest (HDBR) on core body temperature rhythm.

Parameter	*Site*	3rd week	8th week
Mesor	*BBR2-2*	36.73 [36.42, 37.10]	36.81 [36.60, 37.13]
	*Cocktail*	36.87 [36.77, 36.96]	36.91 [36.82, 37.02]
	*BBR2-2* + *Cocktail*	36.82 [36.66, 36.97]	36.88 [36.76, 37.01]
Amplitude	*BBR2-2*	0.52 [0.40, 0.63]	0.55 [0.47, 0.65]
	*Cocktail*	0.48 [0.40, 0.57]	0.54 [0.44, 0.63]
	*BBR2-2* + *Cocktail*	0.50 [0.43, 0.57]	0.54 [0.47, 0.61]
Acrophase	*BBR2-2*	16.44 [15.47, 17.51]	17.07 [16.40, 17.90]
	*Cocktail*	16.11 [15.43, 16.71]	16.43 [15.79, 16.99]
	*BBR2-2* + *Cocktail*	16.23 [15.68, 16.79]	16.68 [16.16, 17.16]

Circadian parameters were determined by cosinor analysis with a 24-h cycle. Data were collected during the 3rd and the 8th week of two HDBR studies (*BBR2-2*: *n* = 5, *Cocktail*: *n* = 8). Data are means and their nonparametric bootstrapped 95% CIs.

**Table 3:** The effects of *Time*, *Site*, and their interaction on circadian parameters mesor, amplitude, and acrophase.

Parameter	Effect	*F*_1,11_	*p*	Effect size *g* [95% CI]
Mesor	*Time*	1.71	0.217	0.34 [−0.18, 0.82]
	*Site*	0.64	0.441	0.43 [−0.63, 1.78]
	*Time* × *Site*	0.13	0.722	−0.19 [−1.40, 0.89]
Amplitude	*Time*	1.71	0.217	0.39 [−0.17, 0.95]
	*Site*	0.10	0.759	−0.16 [−0.95, 0.61]
	*Time* × *Site*	0.13	0.728	0.19 [−0.94, 1.21]
Acrophase	*Time*	12.10	0.005	0.85 [ 0.15, 1.51]
	*Site*	0.72	0.416	−0.46 [−1.25, 0.35]
	*Time* × *Site*	1.31	0.278	−0.61 [−1.64, 0.71]

Two-way ANOVA was performed using *Time* (3rd week, 8th week) as a within-subject factor and Site (*BBR2-2*, *Cocktail*) as a between-subject factor. *F*, F-statistic; *p*, *p*-value. Effect size is Hedges’s *g* and their bootstrapped 95% CI.

## Discussion

We investigated the effect of HDBR on the circadian rhythm of CBT in healthy men. Data were collected as part of two independent bed rest studies conducted at different sites. We observed a significant phase delay of CBT from the 3rd to the 8th week of HDBR. Moreover, this effect was comparable across study sites, suggesting a robust effect of bed rest *per se* on the circadian timing system.

Our findings are in line with previous short-term bed rest studies (≤17 days). Samel et al. (1993) reported a phase delay of body temperature by 18 min during 7-day HDBR. Monk et al. (1997) assessed changes in rectal temperature in a 17-day HDBR study. At the end of bed rest (days 15–17), the phase of rectal temperature rhythm in seven men was delayed by approximately 40 min compared to days 5–7. Similar findings were found for melatonin secretion profiles. Following an 11-day HDBR study, the peak of melatonin secretion was delayed by 16 min (Hurwitz et al., 2004). They performed the initial data collection within the first days of HDBR (≤5 days) and investigated the effects of short periods of bed rest (≤12 days), resulting in a phase delay between ~1.3 and 4.0 min/day. In addition, we recently published data from the *BBR2-2* study showing that the circadian phase of rectal CBT is delayed by on average of 1 min/day during 50 days of bed rest (Mendt et al., 2021). It can be speculated that the effects observed during the first weeks of HDBR are related to the initial psychophysiological adaptations during bed rest. Psychological stress markers are significantly increased after one week of HDBR compared to before bed rest (Rai and Kaur, 2011). Pains, aches, symptoms of discomfort, and sleep issues especially during the first week of HDBR, are often reported by participants (Meck et al., 2009). In the present experiments, however, the first data collection was performed after a minimum of 19 days of HDBR. The timing of these data collections argues against significant moderating and/or confounding effects on circadian rhythms related to sleep and psychophysiological stressors, and suggests that immobilization and reduced physical activity weakened the entrainment of the circadian system to the 24-h period.

In both experiments, we used a non-invasive method for monitoring CBT. The average phase delay was considerably larger in the *BBR2-2* experiment compared to the *Cocktail* experiment (1.4 vs. 0.6 min/day). We attribute this difference to inter-individual responses to long-duration bed rest and potential sampling bias due to the small sample sizes. This notion is also supported by the visual inspection of individual data points. As shown in Figure 1C, the difference was substantially driven by a single participant of the *BBR2-2* experiment (*BBR2-2* participant whose rhythm phase occurred earliest in the 3rd week). Excluding the data of this participant decreased the average phase delay from 1.4 to 0.9 min/day. As part of *BBR2-2*, we also acquired temperature data using a rectal probe. These data were published elsewhere (Mendt et al., 2021). To verify the impact of the aforementioned individual on the point estimate of the phase delay, we examined the circadian rhythms of rectal and heatflux CBT in the subset of the BBR2-2 participants reported in the present paper. We observed similar phase delays irrespective of the CBT measurement (heatflux CBT: 1.4 min/day, rectal CBT: 1.7 min/day). Further, excluding the above-mentioned participant with the extreme phase also led to a significant drop in the circadian phase shift of rectal CBT (heatflux CBT: 0.9 min/day, rectal CBT: 1.1 min/day).

The acrophase in *BBR2-2* was delayed at both data collection points relative to the *Cocktail* experiment. It is possible that this discrepancy is related to the different day-night cycles. Participants in both studies were exposed to a 24-h day-night cycle, but the “day” was shorter and the “night” longer in *BBR2-2* (daytime: 7 am to 11 pm) compared to *Cocktail* (daytime: 6:30 am to 11 pm). Exposure to light levels in the morning (after CBT reaches its minimum) advances the circadian phase, whereas light exposure in the evening (before CBT reaches its minimum) delays the circadian phase (Lack and Wright, 2007). The participants in *Cocktail* were awakened earlier compared to *BBR2-2*, increasing the light exposure in the morning. As a result, the acrophase may have been advanced in the *Cocktail* experiment relative to the *BBR2-2* experiment. Given that the data were not collected at identical time points across the year (e.g., first data collection took place in February for *n* = 7 participants, in July for *n* = 3 participants, and in October for *n* = 3 participants), it is also possible that seasonal effects may have affected our findings (Honma et al., 1992; Meyer et al., 2016; Stothard et al., 2017). We therefore rerun the models and included the days until/since the summer solstice as a covariate. Adjusting the data for the differences of seasons did not affect our findings (*p* = 0.008).

Notably, the acrophase of CBT was delayed in both *BBR2-2* and *Cocktail* participants despite highly comparable experimental conditions and procedures. External cues in the present experiments such as strict sleep-wake/rest-activity cycles, regular day-night cycles [participants were exposed to daylight illumination corresponding to standard indoor light levels (i.e., 100–500 lux)], and regular mealtimes are all well-known critical “zeitgeber” to synchronize and entrain the circadian rhythm to the 24-h day-night cycle (Mistlberger and Skene, 2005). Non-photic behavioral “zeitgeber” have the potential to preserve the temporal adaptation of circadian rhythms to the 24-h day irrespective of light levels (Klerman et al., 1998; Wright et al., 2001). For instance, in a study employing light levels as low as 1.5 lux, the entrainment to a 24-h day could be maintained by scheduled sleep/wake (rest/activity) cycles (Wright et al., 2001). Yet, irrespective of these various non-photic and photic cues, our and previous data from HDBR studies observed a phase delay. Given that HDBR studies employed standard indoor light levels and followed strict 24-h day/night (sleep/wake) cycles, we attribute the observed phase delays to bed rest rather than to the effects of altered light levels and/or non-photic cues.

Although, we observed a phase delay of the circadian rhythm of CBT in two standardized and independent HDBR studies our data are not without limitations. First, the use of short-wavelength enriched light-emitting technologies such as laptops or smartphones was not restricted (except during night). Short-wavelength light has been shown to induce a phase shift of circadian rhythms (Chang et al., 2015). Second, our data do not allow any conclusions about the effects relative to before HDBR or recovery after the cessation of HDBR. We also did not collect any physiological or neurobehavioral data to identify the relevance of the observed phase shifts. Investigation of the acute effects of horizontal bed rest (HBR) and HDBR on sleep revealed a decrease in slow-wave sleep with HDBR (Komada et al., 2006; Boschert et al., 2019). Data from long-duration bed rest studies showed that slow-wave sleep and total sleep time decreased after 21 days in HBR and HDBR (Gkivogkli et al., 2016; Morrison et al., 2017), suggesting that bed rest *per se* rather than tilted posture predominantly accounts for sleep disturbances. Circadian rhythms and sleep are tightly coupled (Van Someren, 2006; Cooper et al., 2018). Circadian shifts are expected to affect sleep, particularly in the presence of fixed sleep/rest cycles prescribed in bed rest studies. This assumption is supported by data from Monk et al. (1997) showing that a phase delay of rectal CBT was associated with a decrease in total sleep time at the end of a 17-day HDBR experiment. In addition to exploring the relationship between circadian disruptions and sleep in future studies, it will also be important to identify the effects of circadian changes on behavior and cognition. Several studies have shown adverse effects of bed rest on brain function and cognitive performance (Lipnicki and Gunga, 2009; Brauns et al., 2019; Friedl-Werner et al., 2020; Basner et al., 2021), mental health (Liu et al., 2012; Stavrou et al., 2018), cardio-vascular changes (Solbiati et al., 2021), and metabolic regulation (Dandanell et al., 2016; Dirks et al., 2016) that can be associated with a lack of sleep (Mullington et al., 2009; Anderson and Bradley, 2013; Goel et al., 2013). To mitigate the adverse neurophysiological and psychological effects associated with circadian disruptions, effective countermeasures are needed to maintain the entrainment of central and peripheral clocks. In addition to melatonin supplementation and lighting interventions (Emens and Burgess, 2015), physical exercise could be a potent countermeasure to mitigate circadian disruptions (Thomas et al., 2020). We recently demonstrated that regular physical activity could counteract phase shifts in response to long-duration bed rest (Mendt et al., 2021). Future studies need to verify the optimal timing, dose, and type of exercise and its combination with lighting protocols to support the entrainment of the circadian timing system during prolonged bed rest.

Taken together, our data demonstrate that bed rest induces a robust phase-delay effect on the circadian rhythm of CBT, as indicated by the average phase delay of about 1 min/day. This finding underlines the significance of physical activity and postural changes for the entrainment of the circadian system and highlights the need for preventive healthcare strategies to mitigate the risk of circadian disruptions when physical activity levels are limited over extended periods of time.

## Data Availability Statement

The data that support the findings of this study are openly available in figshare at: http://doi.org/10.6084/m9.figshare.13633790.

## Ethics Statement

The studies involving human participants were reviewed and approved by the Ethics Committee of Charité – Universitätsmedizin Berlin, Berlin, Germany and the CPP Sud-Ouest et Outre-Mer I, Toulouse, France. The participants provided their written informed consent to participate in this study.

## Author Contributions

DB organized and led the *BBR2-2*. AS and H-CG conceived, designed, planned, and supervised the experiments. AW supported the implementation of the *BBR2-2* experiment. MS and TS collected data of the *BBR2-2* experiment. AF-W supervised and collected the *Cocktail* data with support of KB. SM processed and analyzed the data. SM and AS drafted the manuscript. All authors provided critical feedback, contributed to the interpretation of the results, and approved the final manuscript.

### Conflict of Interest

H-CG was scientific consultant to the company Drägerwerk AG & Co (Lübeck, Germany).

The remaining authors declare that the research was conducted in the absence of any commercial or financial relationships that could be construed as a potential conflict of interest.

## References

[ref1] Allen C. Glasziou P. Del Mar C. (1999). Bed rest: a potentially harmful treatment needing more careful evaluation. Lancet 354, 1229–1233. 10.1016/S0140-6736(98)10063-6, PMID: 10520630

[ref2] Anderson K. N. Bradley A. J. (2013). Sleep disturbance in mental health problemsand neurodegenerative disease. Nat. Sci. Sleep 5, 61–75. 10.2147/NSS.S34842, PMID: 23761983 PMC3674021

[ref3] Arc-Chagnaud C. Py G. Fovet T. Roumanille R. Demangel R. Pagano A. F.. (2020). Evaluation of an antioxidant and anti-inflammatory cocktail against human hypoactivity-induced skeletal muscle deconditioning. Front. Physiol. 11:71. 10.3389/fphys.2020.00071, PMID: 32116779 PMC7028694

[ref4] Atkinson G. Edwards B. Reilly T. Waterhouse J. (2007). Exercise as a synchroniser of human circadian rhythms: an update and discussion of the methodological problems. Eur. J. Appl. Physiol. 99, 331–341. 10.1007/s00421-006-0361-z, PMID: 17165050

[ref5] Baron K. G. Reid K. J. (2014). Circadian misalignment and health. Int. Rev. Psychiatry 26, 139–154. 10.3109/09540261.2014.911149, PMID: 24892891 PMC4677771

[ref6] Basner M. Dinges D. F. Howard K. Moore T. M. Gur R. C. Mühl C.. (2021). Continuous and intermittent artificial gravity as a countermeasure to the cognitive effects of 60 days of head-down tilt bed rest. Front. Physiol. 12:643854. 10.3389/fphys.2021.643854, PMID: 33815148 PMC8009974

[ref7] Belavý D. L. Bock O. Börst H. Armbrecht G. Gast U. Degner C.. (2010). The 2nd Berlin BedRest study: protocol and implementation. J. Musculoskelet. Neuronal Interact. 10, 207–219. PMID: 20811145

[ref8] Boschert A. L. Elmenhorst D. Gauger P. Li Z. Garcia-Gutierrez M. T. Gerlach D.. (2019). Sleep is compromised in −12° head down tilt position. Front. Physiol. 10:397. 10.3389/fphys.2019.00397, PMID: 31040791 PMC6477049

[ref9] Brauns K. Werner A. Gunga H. C. Maggioni M. A. Dinges D. F. Stahn A. (2019). Electrocortical evidence for impaired affective picture processing after long-term immobilization. Sci. Rep. 9:16610. 10.1038/s41598-019-52555-1 31719552 PMC6851182

[ref10] Chang A. M. Aeschbach D. Duffy J. F. Czeisler C. A. (2015). Evening use of light-emitting eReaders negatively affects sleep, circadian timing, and next-morning alertness. Proc. Natl. Acad. Sci. U. S. A. 112, 1232–1237. 10.1073/pnas.1418490112, PMID: 25535358 PMC4313820

[ref11] Cooper J. M. Halter K. A. Prosser R. A. (2018). Circadian rhythm and sleep-wake systems share the dynamic extracellular synaptic milieu. Neurobiol. Sleep Circadian Rhythm. 5, 15–36. 10.1016/j.nbscr.2018.04.001, PMID: 31236509 PMC6584685

[ref13] Cornelissen G. (2014). Cosinor-based rhythmometry. Theor. Biol. Med. Model. 11:16. 10.1186/1742-4682-11-16, PMID: 24725531 PMC3991883

[ref14] Crowley S. J. Cain S. W. Burns A. C. Acebo C. Carskadon M. A. (2015). Increased sensitivity of the circadian system to light in early/mid-puberty. J. Clin. Endocrinol. Metab. 100, 4067–4073. 10.1210/jc.2015-2775, PMID: 26301944 PMC4702443

[ref15] Dandanell S. Oberholzer L. Keiser S. Andersen A. B. Haider T. Hilty M. P.. (2016). Effect of alterations in blood volume with bed rest on glucose tolerance. J. Appl. Physiol. 121, 1098–1105. 10.1152/japplphysiol.00624.2016, PMID: 27633742

[ref16] Dibner C. (2020). The importance of being rhythmic: living in harmony with your body clocks. Acta Physiol. 228:e13281. 10.1111/apha.13281 30980501

[ref17] Dirks M. L. Wall B. T. Van De Valk B. Holloway T. M. Holloway G. P. Chabowski A.. (2016). One week of bed rest leads to substantial muscle atrophy and induces whole-body insulin resistance in the absence of skeletal muscle lipid accumulation. Diabetes 65, 2862–2875. 10.2337/db15-1661, PMID: 27358494

[ref18] Emens J. S. Burgess H. J. (2015). Effect of light and melatonin and other melatonin receptor agonists on human circadian physiology. Sleep Med. Clin. 10, 435–453. 10.1016/j.jsmc.2015.08.001, PMID: 26568121 PMC4648706

[ref19] Friedl-Werner A. Brauns K. Gunga H. C. Kühn S. Stahn A. C. (2020). Exercise-induced changes in brain activity during memory encoding and retrieval after long-term bed rest. NeuroImage 223:117359. 10.1016/j.neuroimage.2020.117359, PMID: 32919056

[ref20] Gkivogkli P. Frantzidis C. Karagianni M. Rosenzweig I. Papadeli C. Bamidis P. (2016). “Sleep Macro-Architecture Disturbances During a 60 Days 60 Head Down Tilt Bed-Rest and the Effect of Sledge Jumping System (SJS) as a Countermeasure to Prevent Those Changes” in SAN2016 Meeting; October 6–9, 2016; Corfu, Greece.

[ref21] Goel N. Basner M. Rao H. Dinges D. F. (2013). “Chapter Seven - Circadian Rhythms, Sleep Deprivation, and Human Performance,” in Progress in Molecular Biology and Translational Science. ed. Gillette M. U. (Academic Press), 155–190. 10.1016/B978-0-12-396971-2.00007-5 PMC3963479 23899598

[ref22] Gunga H. C. Sandsund M. Reinertsen R. E. Sattler F. Koch J. (2008). A non-invasive device to continuously determine heat strain in humans. J. Therm. Biol. 33, 297–307. 10.1016/j.jtherbio.2008.03.004

[ref23] Hargens A. R. Vico L. (2016). Long-duration bed rest as an analog to microgravity. J. Appl. Physiol. 120, 891–903. 10.1152/japplphysiol.00935.2015, PMID: 26893033

[ref24] Honma K. I. Honma S. Kohsaka M. Fukuda N. (1992). Seasonal variation in the human circadian rhythm: dissociation between sleep and temperature rhythm. Am. J. Phys. Regul. Integr. Comp. Phys. 262, R885–R891. 10.1152/ajpregu.1992.262.5.r885 1590482

[ref25] Hurwitz S. Cohen R. J. Williams G. H. (2004). Diurnal variation of aldosterone and plasma renin activity: timing relation to melatonin and cortisol and consistency after prolonged bed rest. J. Appl. Physiol. 96, 1406–1414. 10.1152/japplphysiol.00611.2003, PMID: 14660513

[ref26] James S. M. Honn K. A. Gaddameedhi S. Van Dongen H. P. A. (2017). Shift work: disrupted circadian rhythms and sleep—implications for health and well-being. Curr. Sleep Med. Rep. 3, 104–112. 10.1007/s40675-017-0071-6, PMID: 29057204 PMC5647832

[ref27] Kimberger O. Saager L. Egan C. Sanchez I. P. Dizili S. Koch J.. (2013). The accuracy of a disposable noninvasive core thermometer. Can. J. Anesth. 60, 1190–1196. 10.1007/s12630-013-0047-z, PMID: 24214518

[ref28] Kimberger O. Thell R. Schuh M. Koch J. Sessler D. I. Kurz A. (2009). Accuracy and precision of a novel non-invasive core thermometer. Br. J. Anaesth. 103, 226–231. 10.1093/bja/aep134, PMID: 19482858

[ref29] Kirby K. N. Gerlanc D. (2013). BootES: an R package for bootstrap confidence intervals on effect sizes. Behav. Res. Methods 45, 905–927. 10.3758/s13428-013-0330-5, PMID: 23519455

[ref30] Klerman E. B. Rimmer D. W. Dijk D. J. Kronauer R. E. Rizzo J. F. Czeisler C. A. (1998). Nonphotic entrainment of the human circadian pacemaker. Am. J. Phys. Regul. Integr. Comp. Phys. 274, R991–R996. 10.1152/ajpregu.1998.274.4.r991 9575961

[ref31] Komada Y. Mizuno K. Mishima K. Sato H. Inoue Y. Tanaka H.. (2006). Effects of acute simulated microgravity on nocturnal sleep, daytime vigilance, and psychomotor performance: comparison of horizontal and 6° head-down bed rest. Percept. Mot. Skills 103, 307–317. 10.2466/PMS.103.2.307-317 17165393

[ref32] Korompeli A. Muurlink O. Kavrochorianou N. Katsoulas T. Fildissis G. Baltopoulos G. (2017). Circadian disruption of ICU patients: a review of pathways, expression, and interventions. J. Crit. Care 38, 269–277. 10.1016/j.jcrc.2016.12.006, PMID: 28012425

[ref33] Lack L. C. Wright H. R. (2007). Chronobiology of sleep in humans. Cell. Mol. Life Sci. 64, 1205–1215. 10.1007/s00018-007-6531-2, PMID: 17364140 PMC11136055

[ref34] Lewis P. Korf H. W. Kuffer L. Groß J. V. Erren T. C. (2018). Exercise time cues (zeitgebers) for human circadian systems can foster health and improve performance: a systematic review. BMJ Open Sport Exerc. Med. 4:e000443. 10.1136/bmjsem-2018-000443, PMID: 30687511 PMC6330200

[ref35] Lewis P. Oster H. Korf H. W. Foster R. G. Erren T. C. (2020). Food as a circadian time cue — evidence from human studies. Nat. Rev. Endocrinol. 16, 213–223. 10.1038/s41574-020-0318-z, PMID: 32055029

[ref36] Lipnicki D. M. Gunga H. C. (2009). Physical inactivity and cognitive functioning: results from bed rest studies. Eur. J. Appl. Physiol. 105, 27–35. 10.1007/s00421-008-0869-5, PMID: 18797919

[ref37] Liu Q. Zhou R. Chen S. Tan C. (2012). Effects of head-down bed rest on the executive functions and emotional response. PLoS One 7:e52160. 10.1371/journal.pone.0052160, PMID: 23284916 PMC3524097

[ref38] McGowan N. M. Uzoni A. Faltraco F. Thome J. Coogan A. N. (2020). The impact of social jetlag and chronotype on attention, inhibition and decision making in healthy adults. J. Sleep Res. 29:e12974. 10.1111/jsr.12974, PMID: 31943451

[ref39] Meck J. V. Dreyer S. A. Warren L. E. (2009). Long-duration head-down bed rest: project overview, vital signs, and fluid balance. Aviat. Space Environ. Med. 80, A01–A08. 10.3357/ASEM.BR01.2009, PMID: 19476163

[ref40] Mendt S. Gunga H. C. Felsenberg D. Belavy D. L. Steinach M. Stahn A. C. (2021). Regular exercise counteracts circadian shifts in core body temperature during long-duration bed rest. NPJ Microgravity. 7:1. 10.1038/s41526-020-00129-1, PMID: 33402671 PMC7785743

[ref41] Mendt S. Maggioni M. A. Nordine M. Steinach M. Opatz O. Belavý D.. (2017). Circadian rhythms in bed rest: monitoring core body temperature via heat-flux approach is superior to skin surface temperature. Chronobiol. Int. 34, 666–676. 10.1080/07420528.2016.1224241, PMID: 27726448

[ref42] Meyer C. Muto V. Jaspar M. Kussé C. Lambot E. Chellappa S. L.. (2016). Seasonality in human cognitive brain responses. Proc. Natl. Acad. Sci. U. S. A. 113, 3066–3071. 10.1073/pnas.1518129113, PMID: 26858432 PMC4801294

[ref43] Mistlberger R. E. Skene D. J. (2004). Social influences on mammalian circadian rhythms: animal and human studies. Biol. Rev. Camb. Philos. Soc. 79, 533–556. 10.1017/S1464793103006353, PMID: 15366762

[ref44] Mistlberger R. E. Skene D. J. (2005). Nonphotic entrainment in humans? J. Biol. Rhythm. 20, 339–352. 10.1177/0748730405277982 16077153

[ref45] Monk T. H. Buysse D. J. Billy B. D. Kennedy K. S. Kupfer D. J. (1997). The effects on human sleep and circadian rhythms of 17 days of continuous bedrest in the absence of daylight. Sleep 20, 858–864. 10.1093/sleep/20.10.858, PMID: 9415945

[ref46] Morrison S. A. Mirnik D. Korsic S. Eiken O. Mekjavic I. B. Dolenc-Groselj L. (2017). Bed rest and hypoxic exposure affect sleep architecture and breathing stability. Front. Physiol. 8:410. 10.3389/fphys.2017.00410, PMID: 28676764 PMC5476730

[ref47] Mullington J. M. Haack M. Toth M. Serrador J. M. Meier-Ewert H. K. (2009). Cardiovascular, inflammatory, and metabolic consequences of sleep deprivation. Prog. Cardiovasc. Dis. 51, 294–302. 10.1016/j.pcad.2008.10.003, PMID: 19110131 PMC3403737

[ref48] Oldham M. A. Lee H. B. Desan P. H. (2016). Circadian rhythm disruption in the critically ill: an opportunity for improving outcomes. Crit. Care Med. 44, 207–217. 10.1097/CCM.0000000000001282, PMID: 26308428

[ref49] Pandiarajan M. Hargens A. R. (2020). Ground-based analogs for human spaceflight. Front. Physiol. 11:716. 10.3389/fphys.2020.00716, PMID: 32655420 PMC7324748

[ref50] Papaioannou V. Mebazaa A. Plaud B. Legrand M. (2014). ‘Chronomics’ in ICU: circadian aspects of immune response and therapeutic perspectives in the critically ill. Intensive Care Med. Exp. 2:18. 10.1186/2197-425x-2-18, PMID: 26266918 PMC4513032

[ref12] R Core Team (2019). R: A Language and Environment for Statistical Computing. Available at: http://www.r-project.org

[ref51] Rai B. Kaur J. (2011). Salivary stress markers and psychological stress in simulated microgravity: 21 days in 6° head-down tilt. J. Oral Sci. 53, 103–107. 10.2334/josnusd.53.103, PMID: 21467821

[ref52] Roenneberg T. Merrow M. (2016). The circadian clock and human health. Curr. Biol. 26, R432–R443. 10.1016/j.cub.2016.04.011, PMID: 27218855

[ref53] Samel A. Wegmann H. M. Vejvoda M. (1993). Response of the circadian system to 6° head-down tilt bed rest. Aviat. Sp. Environ. Med. 64, 50–54. PMID: 8424740

[ref54] Soehle M. Dehne H. Hoeft A. Zenker S. (2020). Accuracy of the non-invasive Tcore™ temperature monitoring system to measure body core temperature in abdominal surgery. J. Clin. Monit. Comput. 34, 1361–1367. 10.1007/s10877-019-00430-9, PMID: 31773375

[ref55] Solbiati S. Martin-Yebra A. Vaïda P. Caiani E. G. (2021). Evaluation of cardiac circadian rhythm deconditioning induced by 5-to-60 days of head-down bed rest. Front. Physiol. 11:612188. 10.3389/fphys.2020.612188, PMID: 33519517 PMC7838678

[ref56] Stahn A. C. Werner A. Opatz O. Maggioni M. A. Steinach M. Von Ahlefeld V. W.. (2017). Increased core body temperature in astronauts during long-duration space missions. Sci. Rep. 7:16180. 10.1038/s41598-017-15560-w 29170507 PMC5701078

[ref57] Stavrou N. A. M. Debevec T. Eiken O. Mekjavic I. B. (2018). Hypoxia exacerbates negative emotional state during inactivity: the effect of 21 days hypoxic bed rest and confinement. Front. Physiol. 9:26. 10.3389/fphys.2018.00026, PMID: 29472866 PMC5809445

[ref58] Stothard E. R. McHill A. W. Depner C. M. Birks B. R. Moehlman T. M. Ritchie H. K.. (2017). Circadian entrainment to the natural light-dark cycle across seasons and the weekend. Curr. Biol. 27, 508–513. 10.1016/j.cub.2016.12.041, PMID: 28162893 PMC5335920

[ref59] Sundblad P. Orlov O. Angerer O. Larina I. Cromwell R. (2016). Standardization of bed rest studies in the spaceflight context. J. Appl. Physiol. 121, 348–349. 10.1152/japplphysiol.00089.2016, PMID: 26917693

[ref60] Telias I. Wilcox M. E. (2019). Sleep and circadian rhythm in critical illness. Crit. Care 23:82. 10.1186/s13054-019-2366-0, PMID: 30850003 PMC6408803

[ref61] Thomas J. M. Kern P. A. Bush H. M. McQuerry K. J. Black W. S. Clasey J. L.. (2020). Circadian rhythm phase shifts caused by timed exercise vary with chronotype. JCI Insight. 5:e134270. 10.1172/jci.insight.134270, PMID: 31895695 PMC7098792

[ref62] Thun E. Bjorvatn B. Flo E. Harris A. Pallesen S. (2015). Sleep, circadian rhythms, and athletic performance. Sleep Med. Rev. 23, 1–9. 10.1016/j.smrv.2014.11.003, PMID: 25645125

[ref63] Uth M. F. Koch J. Sattler F. (2016). Body core temperature sensing: challenges and new sensor technologies. Procedia Eng. 168, 89–92. 10.1016/j.proeng.2016.11.154

[ref64] Van Someren E. J. W. (2006). Chapter 18: mechanisms and functions of coupling between sleep and temperature rhythms. Prog. Brain Res. 153, 309–324. 10.1016/S0079-6123(06)53018-3 16876583

[ref65] Waterhouse J. Drust B. Weinert D. Edwards B. Gregson W. Atkinson G.. (2005). The circadian rhythm of core temperature: origin and some implications for exercise performance. Chronobiol. Int. 22, 207–225. 10.1081/CBI-200053477 16021839

[ref66] Werner A. Gunga H.-C. (2020). “Monitoring of Core Body Temperature in Humans,” in Stress Challenges and Immunity in Space. ed. Choukèr A. (Cham: Springer International Publishing), 477–498.

[ref67] Wright K. P. Hughes R. J. Kronauer R. E. Dijk D. J. Czeisler C. A. (2001). Intrinsic near-24-h pacemaker period determines limits of circadian entrainment to a weak synchronizer in humans. Proc. Natl. Acad. Sci. U. S. A. 98, 14027–14032. 10.1073/pnas.201530198 11717461 PMC61161

[ref68] Xie Y. Tang Q. Chen G. Xie M. Yu S. Zhao J.. (2019). New insights into the circadian rhythm and its related diseases. Front. Physiol. 10:682. 10.3389/fphys.2019.00682, PMID: 31293431 PMC6603140

